# Comparison of Five Prophylactically Intravenous Drugs in Preventing Opioid-Induced Cough: A Bayesian Network Meta-Analysis of Randomized Controlled Trials

**DOI:** 10.3389/fphar.2021.684276

**Published:** 2021-11-17

**Authors:** Yunxia Dong, Xiaohan Chang

**Affiliations:** ^1^ Department of Anesthesiology, Shengjing Hospital of China Medical University, Shenyang, China; ^2^ Department of Obstetrics and Gynecology, Shengjing Hospital of China Medical University, Shenyang, China

**Keywords:** drugs, pharmacological interventions, network meta-analysis, opioid-induced cough, randomized controlled trials

## Abstract

**Background:** Due to the absence of direct comparisons of different therapeutic drugs in preventing opioid-induced cough (OIC) during the induction of general anesthesia, clinicians often faced difficulties in choosing the optimal drug for these patients. Hence, this network meta-analysis was conducted to solve this problem.

**Methods:** Online databases, including Pubmed, Embase, Web of Science, Cochrane, and Google Scholar, were searched comprehensively to identify eligible randomized controlled trials (RCTs), up to March 15th, 2021. Within a Bayesian framework, network meta-analysis was performed by the “gemtc” version 0.8.2 package of R-3.4.0 software, and a pooled risk ratio (RR) associated with 95% credible interval (CrI) was calculated.

**Results:** A total of 20 RCTs were finally enrolled, and the overall heterogeneity for this study was low to moderate. Traditional pair-wise meta-analysis results indicated that all of the five drugs, namely, lidocaine, ketamine, dezocine, butorphanol, and dexmedetomidine could prevent OIC for four clinical outcomes, compared with the placebo (all *p-values* < 0.05). Moreover, dezocine had the best effect, compared with that of the other drugs (all *p-values* < 0.05). Network meta-analysis results suggested that the top three rank probabilities for four clinical outcomes from best to worst were dezocine, butorphanol, and ketamine based on individual/cumulative rank plots and surface under the cumulative ranking curve (SUCRA) probabilities. The node-splitting method indicated the consistency of the direct and indirect evidence.

**Conclusions:** Our results indicated that all of these five drugs could prevent OIC compared with the placebo. Moreover, the top three rank probabilities for four clinical outcomes from best to worst were dezocine, butorphanol, and ketamine. Our results were anticipated to provide references for guiding clinical research, and further high-quality RCTs were required to verify our findings.

**Systematic Review Registration:** [https://www.crd.york.ac.uk/prospero/], identifier [CRD42021243358].

## Introduction

Due to the rapid onset, short duration, strong analgesia, and reduced cardiovascular response, opioids such as sufentanil, fentanyl, and remifentanil have been widely applied in the induction and maintenance of general anesthesia (Liu et al., 2014; Shuying et al., 2016). However, the complication of opioid-induced cough (OIC) is frequently encountered during the induction of anesthesia, with an incidence rate as high as 65% (Sun et al., 2014). Although most OIC is transient, light, and self-limiting, it is a risk factor for postoperative nausea and vomiting (Peringathara and Robinson, 2016) and is extremely dangerous for patients with comorbidities, such as brain hernia, increased intracranial pressure, increased ocular pressure, open eye injury, pneumothorax, and hypersensitive airway disease (Lin et al., 2005; Yu et al., 2007). Hence, there is an urgent need to take measures to prevent the occurrence of OIC during induction of general anesthesia.

Numerous pharmacological or non-pharmacological measures have been taken to prevent OIC. Therein, non-pharmacological measures are characterized by diluting drug concentration, slowing down injection rate, using the peripheral injection site, reducing the drug dose, instructing patients on performing the huffing maneuver, and verifying the proper administration sequence of the drug (Ambesh et al., 2010; Min et al., 2012; Shrestha et al., 2012; Xiong et al., 2020). Currently, pharmacological interventions have been widely used in the clinical setting, including lidocaine, dezocine, dexmedetomidine, ketamine, and butorphanol (Shuying et al., 2016; Zhang et al., 2018; Clivio et al., 2019). However, most of these articles were compared with the placebo, and direct comparisons of different pharmacological interventions were absent, along with the application of novel drugs. As a result, it was much harder for clinical physicians to choose the optimal therapeutic drug.

As is already known, network meta-analysis could overcome the limitations of traditional meta-analysis and gain evidence directly and indirectly (Lumley, 2002; Zhang et al., 2019). Hence, we applied a Bayesian network meta-analysis of randomized controlled trials (RCTs) to evaluate the efficacy of different pharmacological interventions for OIC. In this article, five different therapeutic drugs, namely, lidocaine, ketamine, dezocine, butorphanol, and dexmedetomidine were finally enrolled. Four clinical outcomes comprising incidence of OIC, mild severity of OIC, moderate severity of OIC, and severe severity of OIC were ultimately evaluated. Our results were anticipated to provide some references for guiding clinical practice.

## Materials and Methods

### Search Strategy

This network meta-analysis was carried out based on the Preferred Reporting Items for Systematic Reviews and Meta-Analysis (PRISMA) guidelines (Page et al., 2021). Online databases, including Pubmed, Embase, Web of Science, Cochrane, and Google Scholar, were searched comprehensively to identify eligible randomized controlled trials (RCTs) up to March 15th, 2021. Our search strategy was mainly comprised three parts utilizing the following keywords in combination with the following Medical Subject Heading (MeSH) terms and text words: “therapeutic drugs”, “lidocaine”, “ketamine”, “dezocine”, “butorphanol”, or “dexmedetomidine” and “opioid-induced cough”, “sufentanil-induced cough”, “fentanyl-induced cough”, or “remifentanil-induced cough” and (“randomized controlled trials”). Additional articles were manually screened from the reference lists of eligible studies to avoid omissions. This network meta-analysis was registered in PROSPERO (https://www.crd.york.ac.uk/prospero/) with the registration number “CRD42021243358”.

### Inclusion and Exclusion Criteria

The inclusion criteria in this article were displayed as following: 1) English articles; 2) Randomized controlled trials; 3) At least two of six drugs (lidocaine, ketamine, dezocine, butorphanol, dexmedetomidine, and placebo) should be compared; 4) At least one of four clinical outcomes (incidence of OIC, mild severity of OIC, moderate severity of OIC, and severe severity of OIC) should be evaluated; and 5) Data could be extracted from articles; The exclusion criteria were detailed as follows: 1)Non-English articles; 2) Non-randomized controlled trials; 3) Articles that did not compare at least two of these six drugs; 4) Articles that did not evaluate at least one of four clinical outcomes; and 5) Data could not be extracted from articles;

### Data Extraction

Two blind reviewers independently identified the data of eligible studies, based on our inclusion and exclusion criteria. When any discrepancy existed, we would discuss with a third reviewer to solve this problem. Moreover, we would record the following information for further analysis: the first author’s name of the study, publication year, American Society of Anesthesiologist (ASA) physical status, injection, study type, opioid, treatment, incidence of OIC, mild severity of OIC, moderate severity of OIC, and severe severity of OIC.

### Quality Assessment

In this article, the potential source of bias of eligible RCTs would be evaluated based on the Cochrane Handbook (http://www.cochrane-handbook.org) (Higgins et al., 2019), containing the following seven aspects of bias: 1) Random sequence generation (selection bias); 2) Allocation concealment (selection bias); 3) Blinding of participants and personnel (performance bias); 4) Blinding of outcome assessment (detection bias); 5) Incomplete outcome data (attrition bias); 6) Selective reporting (reporting bias); and 7) Other bias. Finally, each aspect would be graded as a low, high, or unclear risk of bias.

### Statistical Analysis

Within a Bayesian framework, network meta-analysis comprising different therapeutic drugs was performed by the version 0.8.2 “gemtc” package of R software (version 3.4.0; R Foundation, Vienna, Austria) (Lumley, 2002; Valkenhoef and Kuiper, 2016). A non-informative prior distribution was utilized in this Bayesian analysis, and posterior distribution was estimated by Gibbs sampling using the Markov chain Monte Carlo method (Ando et al., 2020; Cha et al., 2021). When three Markov chains run simultaneously, 10,000 simulations and 40,000 iterations were set by us for each chain to calculate the risk ratio (RR) with 95% credible interval (CrI) of model parameters by the mtc.run function. The Brooks–Gelman–Rubin plot, trace plot, and density plot mehods were utilized to assess the model convergence (Wu et al., 2013). Moreover, we would simultaneously obtain the matrix and the plot of rank probabilities, provided by the R package of “gemtc”. When a loop connecting three arms existed, the node-splitting method was utilized to access the inconsistency by reporting its Bayesian *p-*value (Dias et al., 2010; Wang et al., 2018). To evaluate the heterogeneity, the mtc. anohe command of the R package of “gemtc” was utilized by reporting the heterogeneity variance parameter *I* ^*2*^. *I* ^*2*^ > 50% was regarded as significant heterogeneity, and the random effects models would be utilized; otherwise the fixed effect models would be utilized (Ma et al., 2021; Zhou et al., 2021). Sensitivity analysis was also conducted to examine the robustness of our results. In summary, all *p-*values were adopted by a two-sided test, and *p-value* ＜ 0.05 was considered to be statistically significant.

## Results

### Search Results and Study Characteristics

**FIGURE 1:**
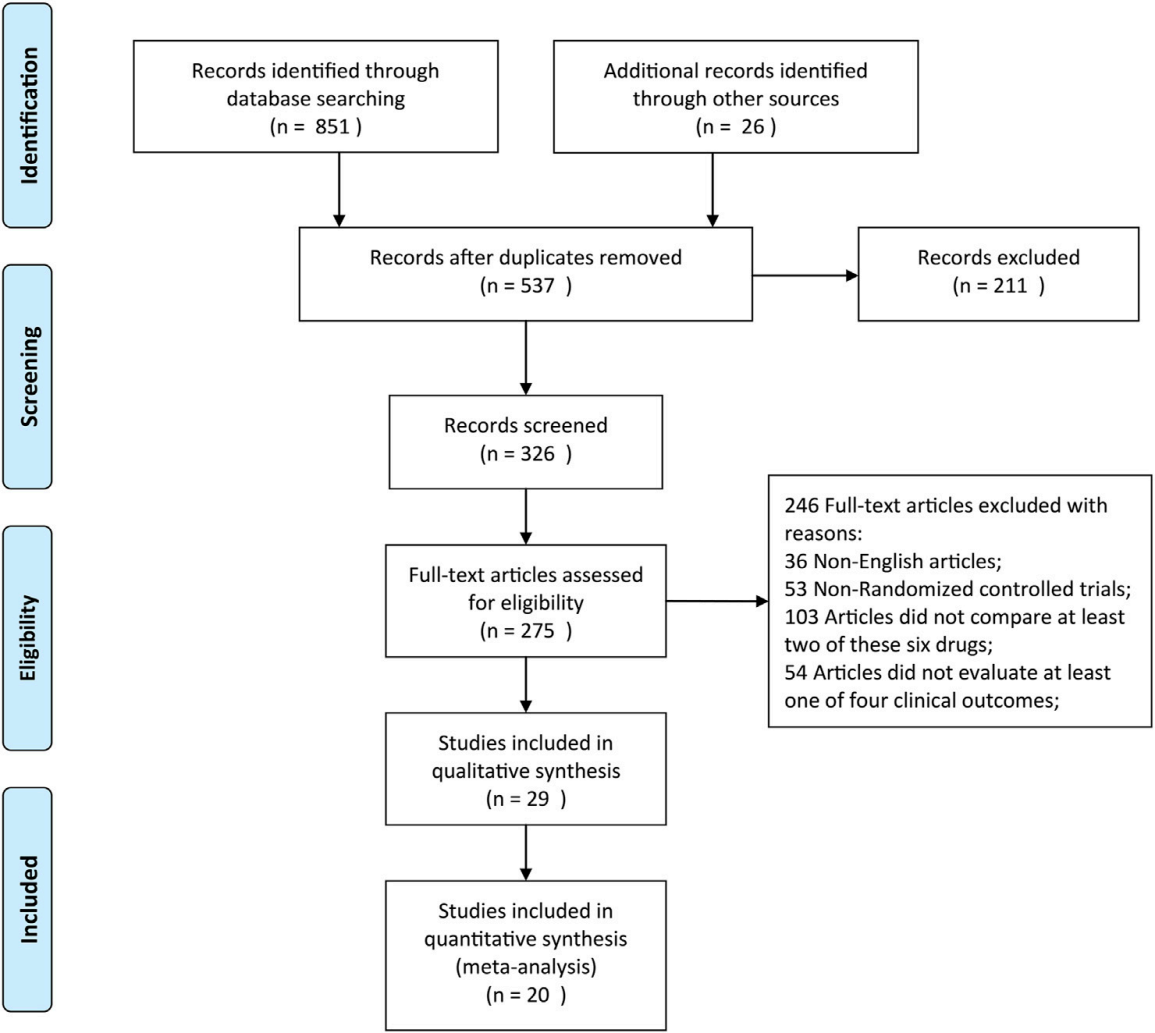
Flow diagram of the literature selection process.

A total of 877 citations were yielded by searching five online databases (Pubmed, Embase, Web of Science, Cochrane, and Google Scholar) by our search strategy. Based on the inclusion and exclusion criteria, 20 RCTs were finally identified and considered eligible for this network meta-analysis (Figure 1) (Lin et al., 2004; Pandey et al., 2004; Pandey et al., 2005; Yeh et al., 2007; Kim et al., 2008; Kim et al., 2009; Bang et al., 2010; Guler et al., 2010; Sun et al., 2011; He et al., 2012; Yu et al., 2012; Gecaj-Gashi et al., 2013; Honarmand et al., 2013; Sun and Huang, 2013; Saleh et al., 2014; Liu et al., 2015; Cheng et al., 2016; Naldan et al., 2019; Yin and Zhang, 2019; Zhou et al., 2019). Moreover, Table 1 summarizes the detailed information of individual studies enrolled in this network meta-analysis. As for quality assessment, all of the 20 RCTs were evaluated based on the Cochrane Handbook (http://www.cochrane-handbook.org) and graded each potential source of bias as low, high, or unclear risk of bias (Supplement Supplementary Figures S1, S2). Moreover, the PRISMA 2020 checklist and PRISMA 2020 for abstract checklist are displayed in Supplementary Tables S1, S2, respectively.

**TABLE 1:** Detailed information of individual studies enrolled in this network meta-analysis.

Study	Year	ASA	Injection	Study type	Opioid	Treatment	Incidence of OIC		Mild severity of OIC		Moderate severity of OIC		Severe severity of OIC	
							Responders	Sample size	Responders	Sample size	Responders	sampleSize	Responders	sampleSize
Yin	2019	I-II	Intravenous	RCT	sufentanil	placebo	33	40	5	40	16	40	12	40
						butorphanol	0	80	0	80	0	80	0	80
Zhou	2019	I-II	Intravenous	RCT	fentanyl	placebo	22	42	8	42	8	42	6	42
						dexmedetomidine	29	126	16	126	8	126	5	126
Naldan	2019	I	Intravenous	RCT	fentanyl	placebo	16	40	7	40	6	40	2	40
						lidocaine	6	40	4	40	2	40	0	40
Cheng	2016	I-II	Intravenous	RCT	fentanyl	placebo	33	105	20	105	7	105	6	105
						butorphanol	16	210	15	210	1	210	0	210
Liu	2015	I-II	Intravenous	RCT	sufentanil	placebo	59	185	13	185	21	185	25	185
						dezocine	0	185	0	185	0	185	0	185
Saleh	2014	I-II	Intravenous	RCT	fentanyl	placebo	53	100	25	100	17	100	11	100
						ketamine	20	100	10	100	6	100	4	100
						dexmedetomidine	34	100	16	100	11	100	7	100
Gecaj-Gashi	2013	I-II	Intravenous	RCT	fentanyl	placebo	27	62	19	62	5	62	3	62
						lidocaine	24	124	18	124	5	124	1	124
Honarmand	2013	I-II	Intravenous	RCT	remifentanil	placebo	17	30	8	30	8	30	1	30
						ketamine	6	30	4	30	2	30	0	30
Sun	2013	I-II	Intravenous	RCT	sufentanil	placebo	16	60	6	60	5	60	5	60
						dexmedetomidine	11	180	4	180	3	180	4	180
He	2012	I-II	Intravenous	RCT	fentanyl	placebo	61	100	30	100	23	100	8	100
						dexmedetomidine	58	200	29	200	22	200	7	200
Yu	2012	I-II	Intravenous	RCT	fentanyl	placebo	45	110	21	110	13	110	11	110
						dexmedetomidine	25	110	10	110	7	110	8	110
Sun	2011	I-II	Intravenous	RCT	fentanyl	placebo	42	60	NA	NA	NA	NA	NA	NA
						dezocine	0	60	NA	NA	NA	NA	NA	NA
Guler	2010	I-II	Intravenous	RCT	fentanyl	placebo	23	100	10	100	12	100	1	100
						lidocaine	11	100	7	100	4	100	0	100
						ketamine	0	100	0	100	0	100	0	100
Bang	2010	I-II	Intravenous	RCT	remifentanil	placebo	24	79	17	79	5	79	2	79
						lidocaine	20	79	7	79	6	79	7	79
Kim	2009	I-II	Intravenous	RCT	remifentanil	placebo	43	154	23	154	12	154	8	154
						ketamine	18	156	10	156	4	156	4	156
Kim	2008	I-II	Intravenous	RCT	remifentanil	placebo	69	250	33	250	21	250	15	250
						lidocaine	38	250	22	250	10	250	6	250
Yeh	2007	I-II	Intravenous	RCT	fentanyl	placebo	39	180	26	180	9	180	4	180
						ketamine	13	180	9	180	4	180	0	180
Pandey	2005	I-II	Intravenous	RCT	fentanyl	placebo	28	80	21	80	6	80	1	80
						lidocaine	34	240	22	240	11	240	1	240
Lin	2004	I-II	Intravenous	RCT	fentanyl	placebo	20	31	NA	NA	NA	NA	NA	NA
						lidocaine	4	29	NA	NA	NA	NA	NA	NA
Pandey	2004	I-II	Intravenous	RCT	fentanyl	placebo	86	251	60	251	21	251	5	251
						lidocaine	33	251	23	251	7	251	3	251

OIC: opioid-induced cough; ASA: American Society of Anesthesiologist physical status; RCT: randomized controlled trials; NA: not available.

### Network Structure Diagrams

**FIGURE 2:**
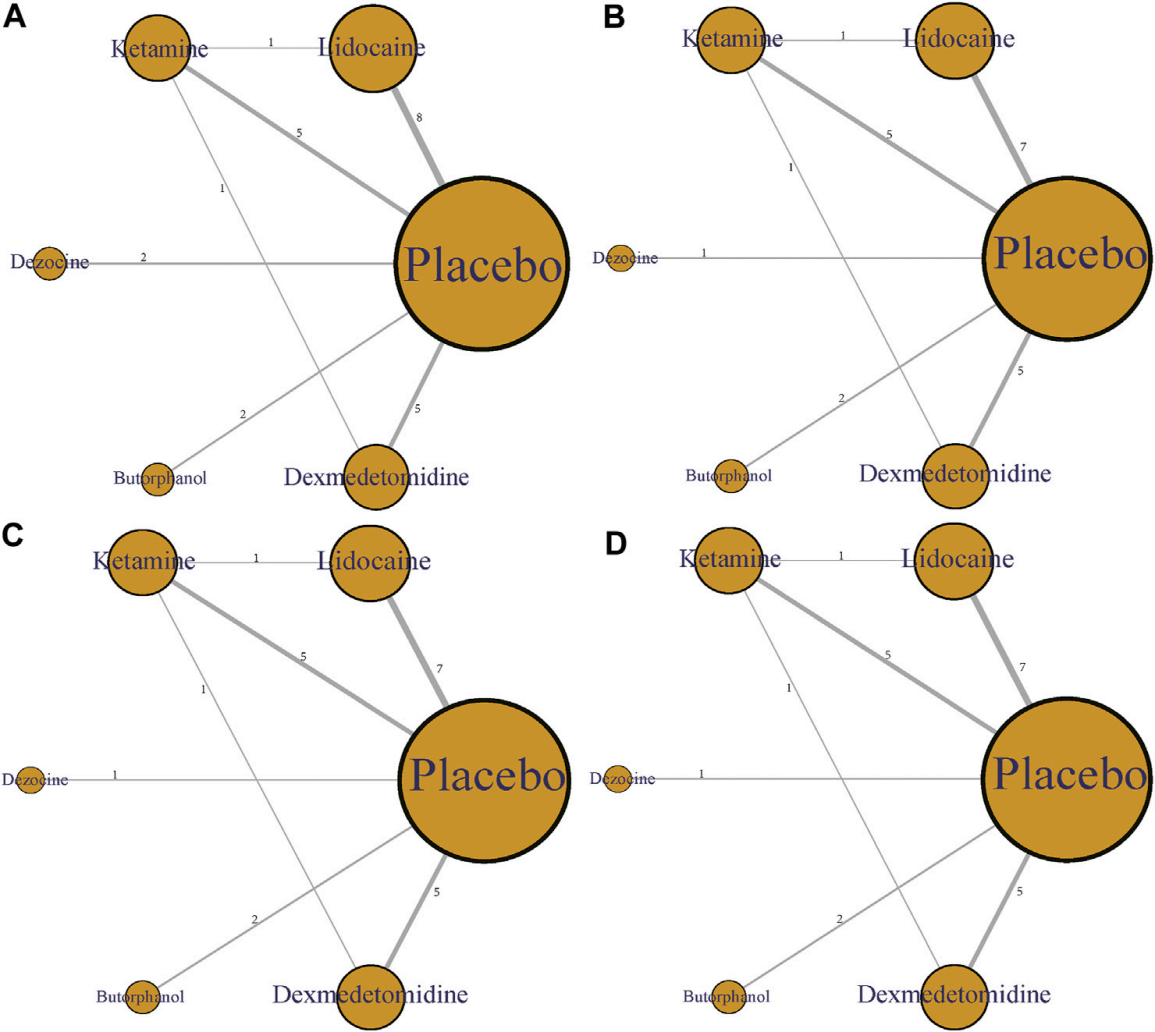
Network structure diagrams. **(A)** Incidence of OIC; **(B)** Mild severity of OIC; **(C)** Moderate severity of OIC; and **(D)** Severe severity of OIC. The numbers showed the number of direct comparisons. Line thicknesses were proportional to the number of direct comparisons. Circle diameters were proportional to the treatment numbers.

In this article, five different therapeutic drugs, namely, lidocaine, ketamine, dezocine, butorphanol, and dexmedetomidine were finally enrolled. Four clinical outcomes comprising incidence of OIC, mild severity of OIC, moderate severity of OIC, and severe severity of OIC were ultimately evaluated. As displayed in Figure 2, the network structure diagrams detailed the direct comparisons between different drugs in the four clinical outcomes, respectively. Besides, the numbers showed the number of direct comparisons. Line thicknesses were proportional to the number of direct comparisons. Circle diameters were proportional to the treatment numbers included in this network meta-analysis.

### Incidence of OIC

**FIGURE 3:**
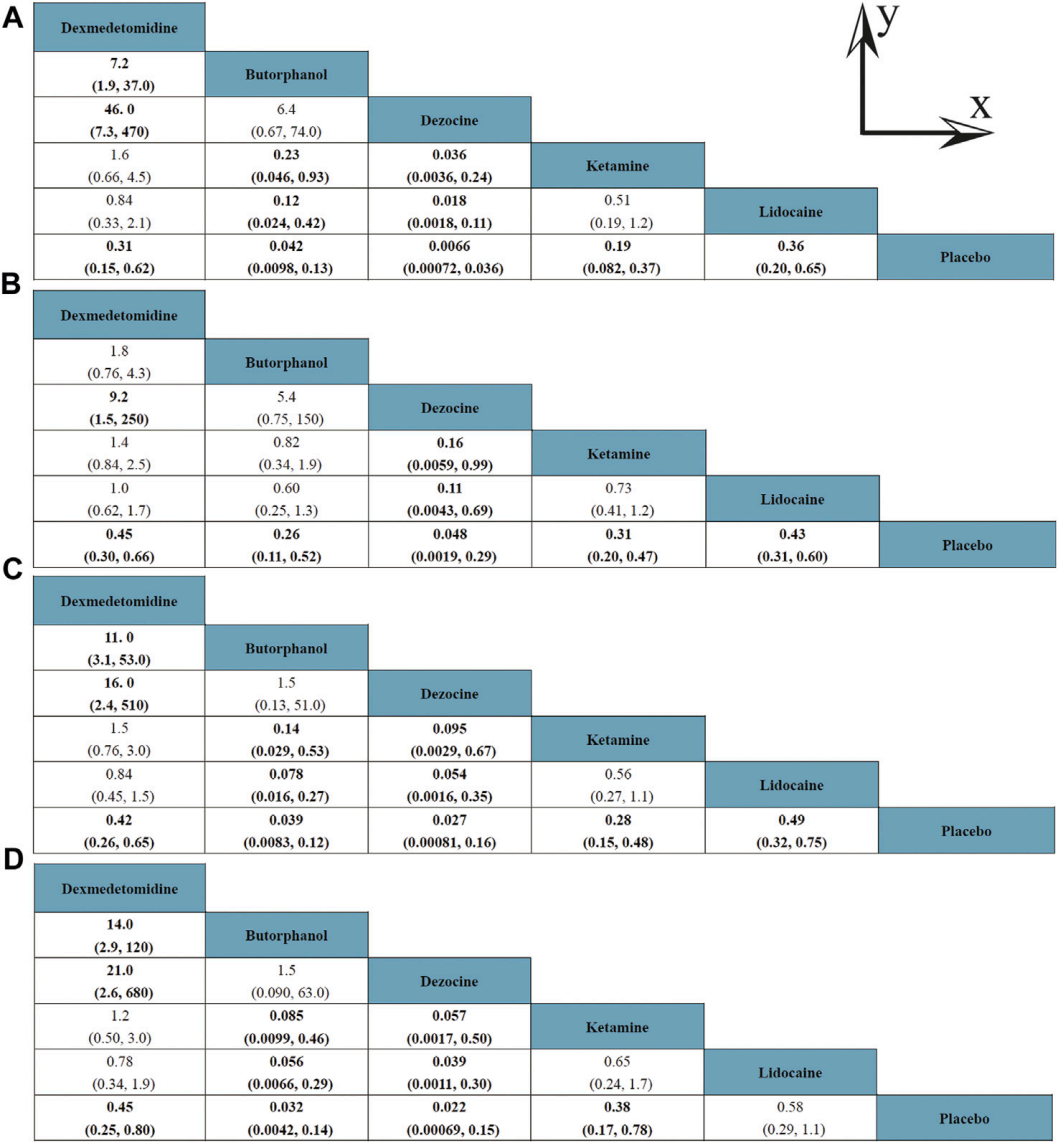
Efficacy of different comparisons of drugs by RRs and corresponding 95% CrIs; **(A)** Incidence of OIC; **(B)** Mild severity of OIC; **(C)** Moderate severity of OIC; and **(D)** Severe severity of OIC. All results were displayed as the ratio of the *Y* axis versus *X* axis. Bold fonts indicated *p-value* < 0.05.

**FIGURE 4:**
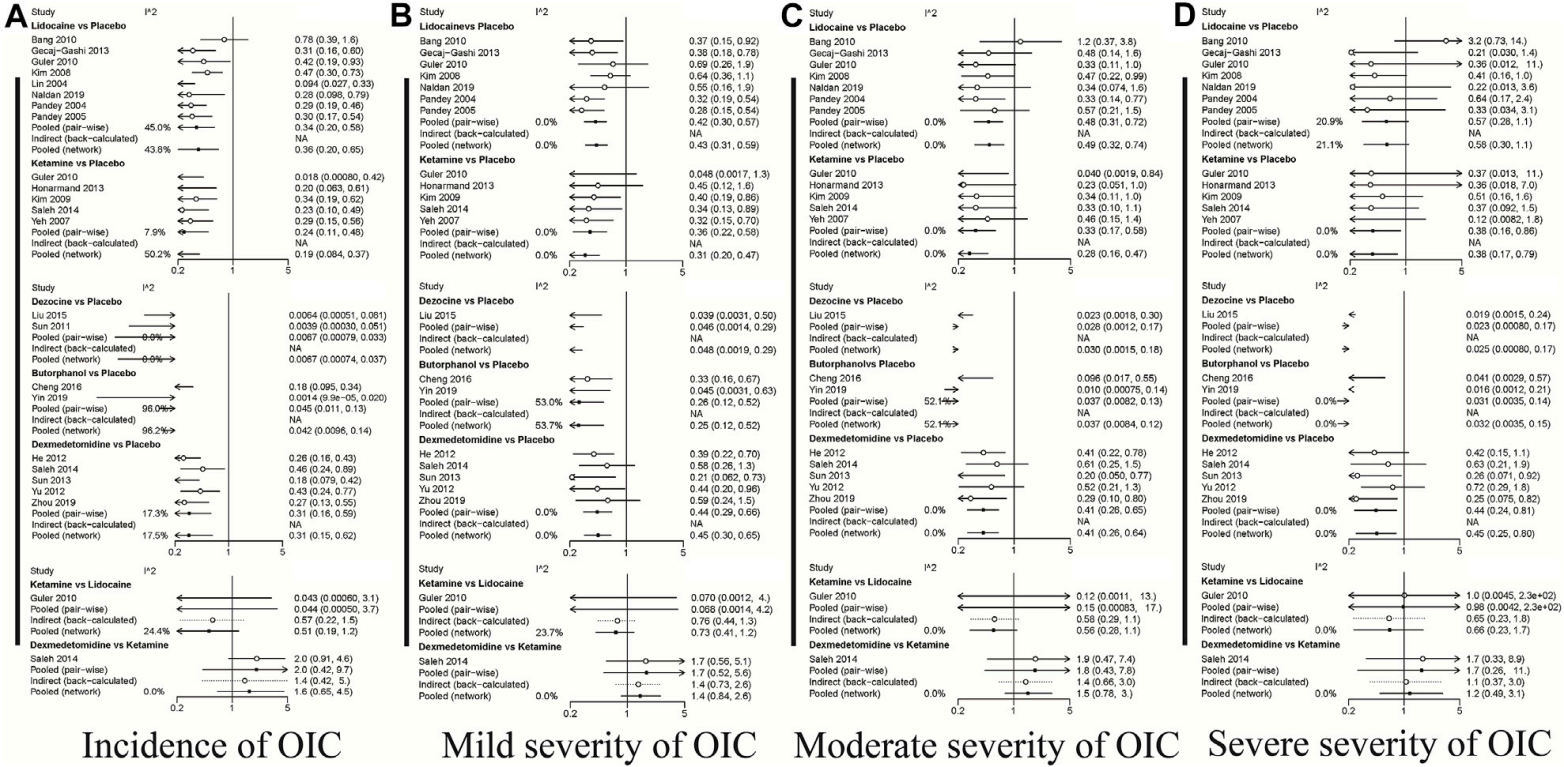
Heterogeneity between different comparisons of drugs. **(A)** Incidence of OIC; **(B)** Mild severity of OIC; **(C)** Moderate severity of OIC; and **(D)** Severe severity of OIC.

**FIGURE 5:**
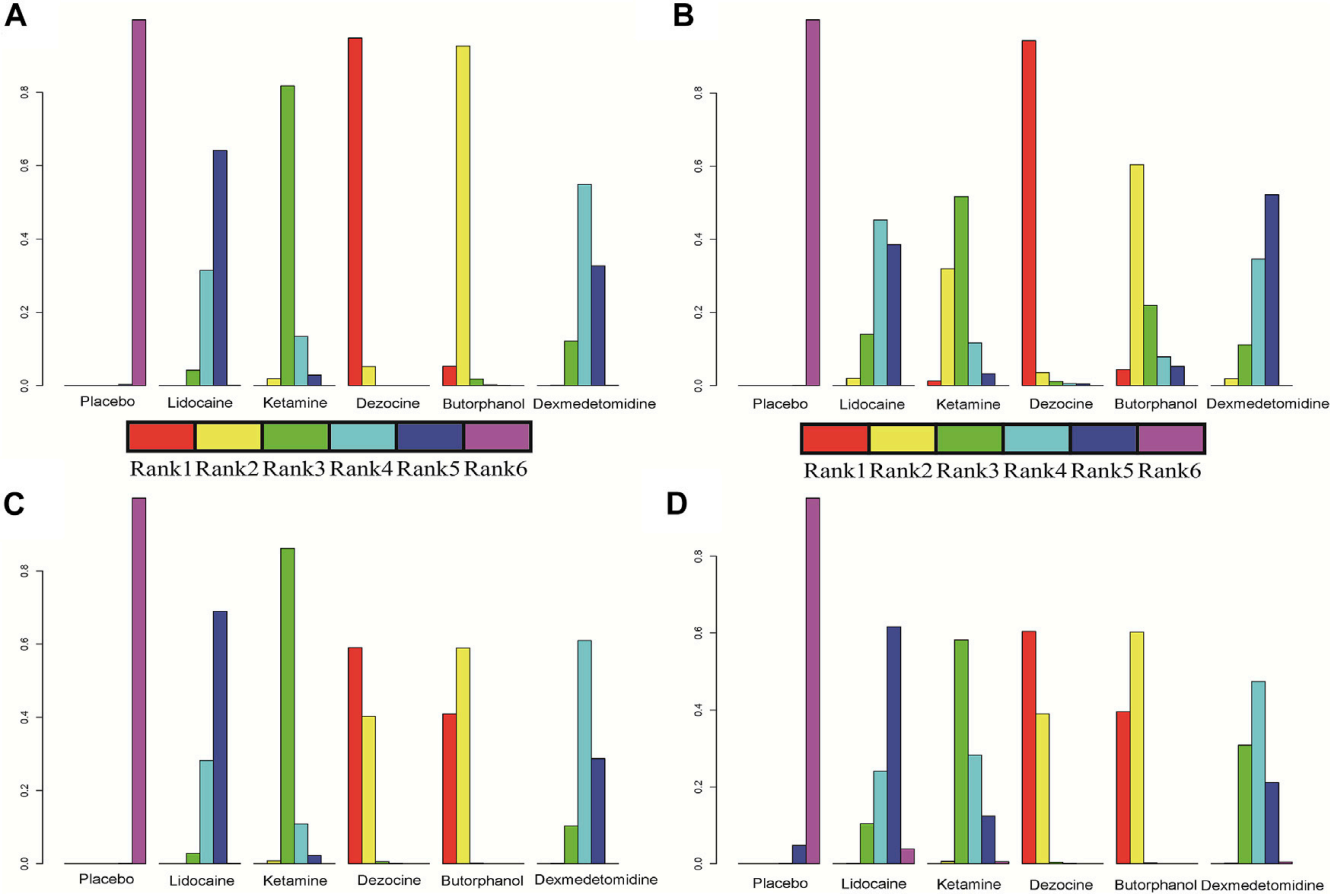
Individual rank plot for four clinical outcomes. **(A)** Incidence of OIC; **(B)** Mild severity of OIC; **(C)** Moderate severity of OIC; and **(D)** Severe severity of OIC.

**FIGURE 6:**
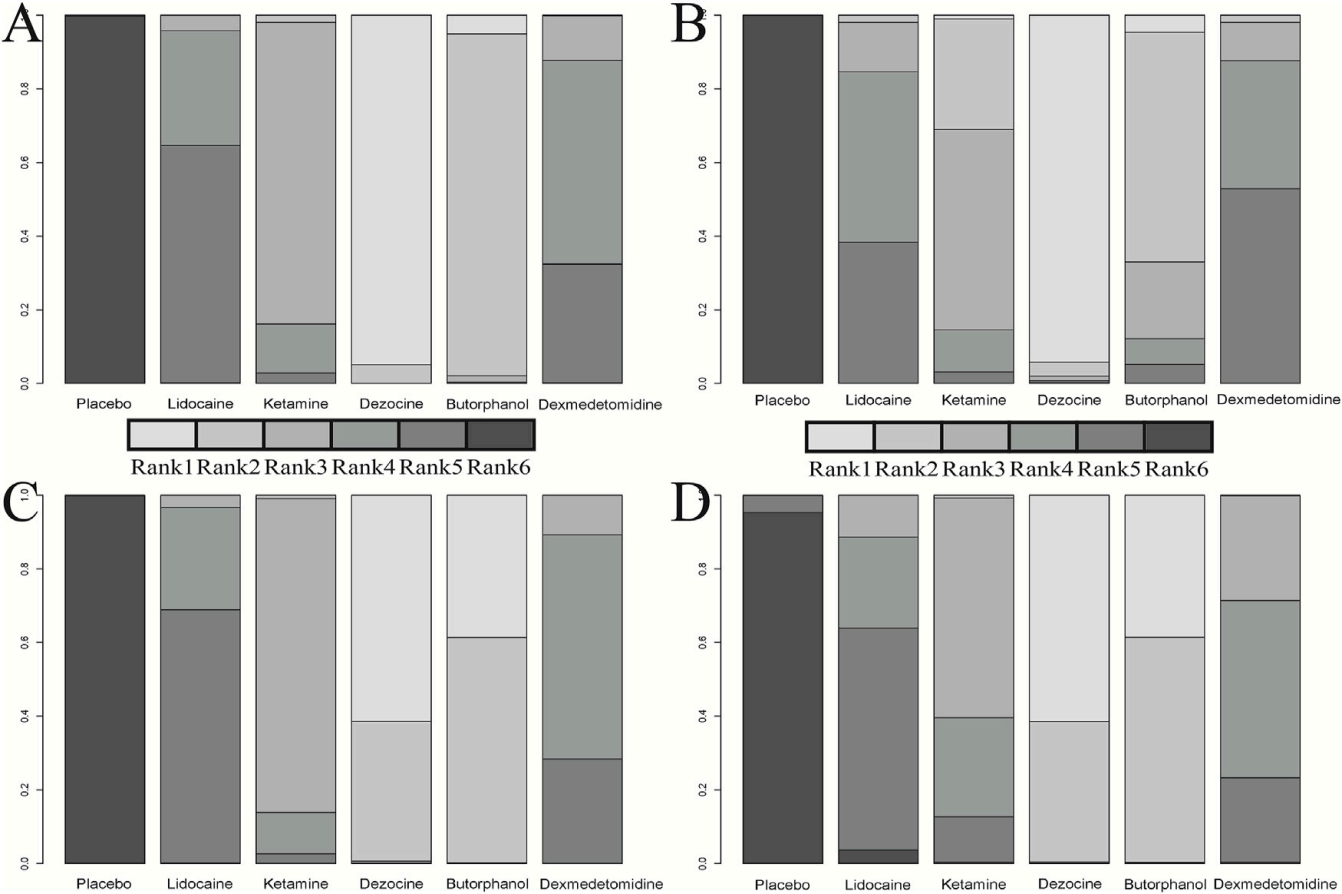
Cumulative rank plot for four clinical outcomes. **(A)** Incidence of OIC; **(B)** Mild severity of OIC; **(C)** Moderate severity of OIC; and **(D)** Severe severity of OIC.

**FIGURE 7:**
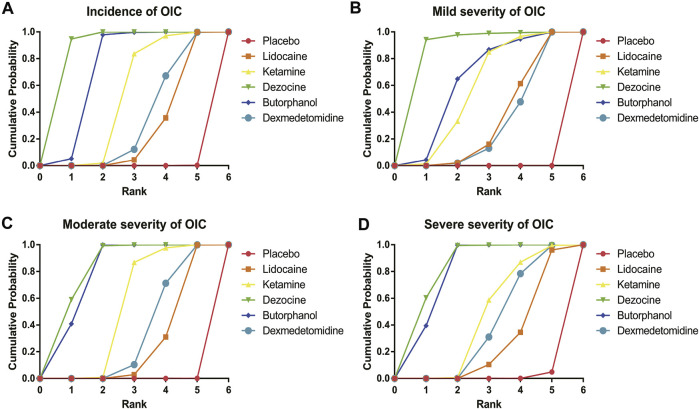
Surface under the cumulative ranking curve (SUCRA) probabilities of different drugs for four clinical outcomes. **(A)** Incidence of OIC; **(B)** Mild severity of OIC; **(C)** Moderate severity of OIC; and **(D)** Severe severity of OIC.

**FIGURE 8:**
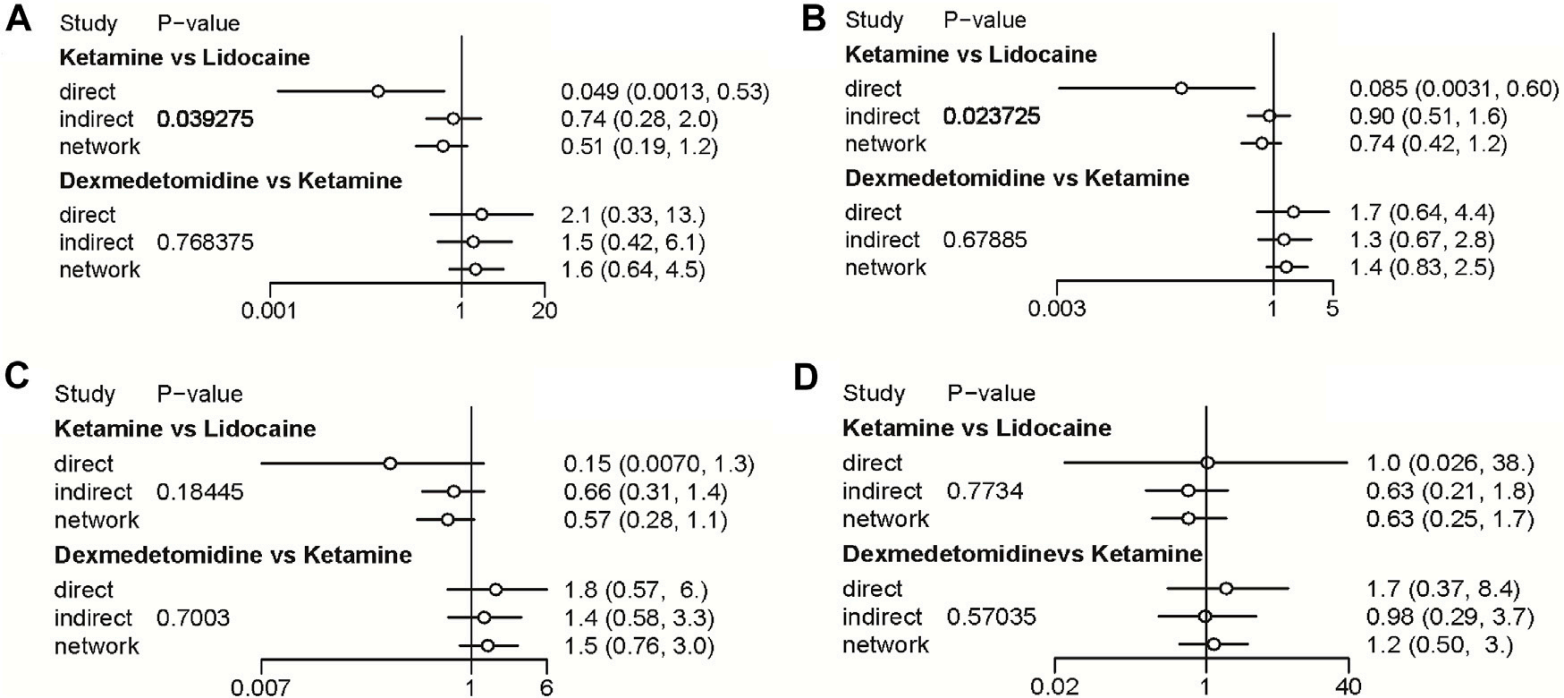
Node-splitting method in comparisons between direct and indirect evidence. **(A)** Incidence of OIC; **(B)** Mild severity of OIC; **(C)** Moderate severity of OIC; and **(D)** Severe severity of OIC.

A total of 20 RCTs, including six drugs (lidocaine, ketamine, dezocine, butorphanol, dexmedetomidine, and placebo) contributed to the clinical outcome of the incidence of OIC. As displayed in Figure 3A and Supplementary Figure S3A, it detailed the efficacy of different comparisons of drugs by RRs and corresponding 95% CrIs. We could easily find that all of the five drugs (lidocaine, ketamine, dezocine, butorphanol, and dexmedetomidine) could prevent the incidence of OIC, compared with the placebo (all *p-values* < 0.05). Moreover, dezocine had the best effect, compared with that of the other drugs (all-values *p* < 0.05). Figure 4A summarizes the heterogeneity between different comparisons of drugs. Individual and cumulative rank plots indicated that the rank probability for the incidence of OIC from best to worst was dezocine, butorphanol, ketamine, dexmedetomidine, lidocaine, and placebo (Figure 5A, Figure 6A). Moreover, their surface under the cumulative ranking curve (SUCRA) probabilities of six drugs for the incidence of OIC are also presented in Figure 7A; Table 2. Additionally, *p-* values of the node-splitting method between ketamine vs lidocaine were below 0.05, indicating the inconsistency of the direct and indirect evidence. *P-values* values of the node-splitting method between dexmedetomidine vs. ketamine were above 0.05, suggesting the consistency of the direct and indirect evidence (Figure 8A). Sensitivity analysis was also conducted as shown in Supplementary Figure S4A, indicating the robustness of our results.

**TABLE 2:** Surface under the cumulative ranking curve (SUCRA) probabilities of different drugs for four clinical outcomes.

Intervention	Placebo (%)	Lidocaine (%)	Ketamine (%)	Dezocine (%)	Butorphanol (%)	Dexmedetomidine (%)
Incidence of OIC	8.39	31.67	55.47	90.77	75.47	38.25
Mild severity of OIC	8.35	38.27	61.05	90.07	66.78	35.48
Moderate severity of OIC	8.35	30.63	55.90	84.70	81.80	38.62
Severe severity of OIC	9.17	31.87	49.32	84.93	81.55	43.18

### Mild Severity of OIC

18 RCTs contributed to the analysis of mild severity of OIC, including six drugs, namely, lidocaine, ketamine, dezocine, butorphanol, dexmedetomidine, and placebo. Figure 3B; Supplementary Figure S3B details the efficacy of different comparisons of drugs by RRs and corresponding 95% CrIs. Similar to previous results, all of the five drugs (lidocaine, ketamine, dezocine, butorphanol, and dexmedetomidine) could reduce mild severity of OIC, compared with that of the placebo (all *p-values* < 0.05). Moreover, dezocine had the best effect compared with that of other drugs (all *p-values* < 0.05). Heterogeneity between different comparisons of drugs is summarized in Figure 4B. Individual and cumulative rank plots explained that the rank probability for mild severity of OIC from first to last was dezocine, butorphanol, ketamine, lidocaine, dexmedetomidine, and placebo (Figure 5B, Figure 6B). Moreover, their surface under the cumulative ranking curve (SUCRA) probabilities of the six drugs for mild severity of OIC are also shown in Figure 7B; Table 2. Besides, *p-*values of the node-splitting method between ketamine vs. lidocaine were below 0.05, indicating the inconsistency of the direct and indirect evidence. *P-*values of the node-splitting method between dexmedetomidine vs. ketamine were more than 0.05, suggesting the consistency of the direct and indirect evidence (Figure 8B). Sensitivity analysis was also conducted as shown in Supplementary Figure S4B, indicating the robustness of our results.

### Moderate Severity of OIC

There were 18 RCTs contributing to the analysis of moderate severity of OIC, including six drugs (lidocaine, ketamine, dezocine, butorphanol, dexmedetomidine, and placebo). Figure 3C; Supplementary Figure S3C detailed the efficacy of different comparisons of drugs by RRs and corresponding 95% CrIs. As same as previous results, all of the five drugs (lidocaine, ketamine, dezocine, butorphanol, and dexmedetomidine) could inhibit the moderate severity of OIC, compared with that of the placebo (all *p-values* < 0.05). Moreover, dezocine had the best effect compared with that of other five drugs (all *p-values* < 0.05). Heterogeneity between different comparisons of drugs is shown in Figure 4C. Similar to the results of incidence of OIC, individual and cumulative rank plots explained that the rank probability for mild severity of OIC from best to worst was dezocine, butorphanol, ketamine, dexmedetomidine, lidocaine, and placebo (Figure 5C, Figure 6C). Furthermore, their surface under the cumulative ranking curve (SUCRA) probabilities of six drugs for moderate severity of OIC are also presented in Figure 7C; Table 2. In addition, ***p-*** values of the node-splitting method were all more than 0.05, indicating the consistency of the direct and indirect evidence (Figure 8C). Sensitivity analysis was also conducted as shown in Supplementary Figure S4C, indicating the robustness of our results.

### Severe Severity of OIC

A total of 18 RCTs contributed to the analysis of severe severity of OIC, including six drugs (lidocaine, ketamine, dezocine, butorphanol, dexmedetomidine, and placebo). Figure 3D; Supplementary Figure S3D detailed the efficacy of different comparisons of drugs by RRs and corresponding 95% CrIs. As same as previous results, four drugs, namely, ketamine, dezocine, butorphanol, and dexmedetomidine could prevent the severe severity of OIC, compared with that of the placebo (all *p-values* < 0.05). Moreover, dezocine had the best effect compared with that of other five drugs (all *p-values* < 0.05). Figure 4D showed the heterogeneity between different comparisons of drugs. Similar to the results of the incidence of OIC, individual and cumulative rank plots explained that the rank probability for severe severity of OIC from first to last was dezocine, butorphanol, ketamine, dexmedetomidine, lidocaine, and placebo (Figure 5D, Figure 6D). Furthermore, the surface under the cumulative ranking curve (SUCRA) probabilities of six drugs for severe severity of OIC are also exhibited in Figure 7D; Table 2. Furthermore, *p-*values of the node-splitting method were all above 0.05, indicating the consistency of the direct and indirect evidence (Figure 8D). Sensitivity analysis was also conducted as shown in Supplementary Figure S4D, indicating the robustness of our results.

## Discussion

Although OIC was a transient, light, and self-limiting disease, it is a well-known adverse effect encountered during opioid administration, and pharmacologically induced cough could even be severe enough to result in death, especially for patients with comorbidities (Tweed and Dakin, 2001; Clivio et al., 2019). Therefore, there was an urgent need to take effective measures for these patients. Currently, pharmacological interventions have been widely used in the clinical setting. Due to the absence of direct comparisons of different pharmacological interventions and the application of novel therapeutic drugs, we, as clinical physicians, often face difficulties in choosing the optimal therapeutic drug for patients for preventing OIC during administration of general anesthesia. Hence, this network meta-analysis of RCTs was conducted to provide a hierarchy of five different therapeutic drugs to provide some references for further clinical research.

In this article, a total of six drugs, namely, lidocaine, ketamine, dezocine, butorphanol, dexmedetomidine, and placebo were finally enrolled. A total of four clinical outcomes comprising incidence of OIC, mild severity of OIC, moderate severity of OIC, and severe severity of OIC were ultimately evaluated. The overall heterogeneity between different comparisons of drugs was low to moderate, except for butorphanol vs placebo. The results of traditional pair-wise meta-analyses indicated that all of the five drugs (lidocaine, ketamine, dezocine, butorphanol, and dexmedetomidine) could prevent OIC for four clinical outcomes, compared with that of the placebo. Moreover, dezocine had the best effect, compared with that of other drugs. Network meta-analysis results suggested that the rank probability for incidence of OIC, moderate severity of OIC, and severe severity of OIC from best to worst was dezocine, butorphanol, ketamine, dexmedetomidine, lidocaine, and placebo, and the rank probability for mild severity of OIC from first to last was dezocine, butorphanol, ketamine, lidocaine, dexmedetomidine, and placebo, according to individual rank plots, cumulative rank plots, and SUCRA probabilities.

Currently, the mechanisms of OIC still remain unclear. Previous studies revealed that two main mechanisms might be among the reasons for OIC. On the one hand, the activation of the parasympathetic nervous system after opioid administration, could result in cough and bronchoconstriction (Yasuda et al., 1978), and on the other hand, the pulmonary chemoreflex could be another possible mechanism, mediated by rapidly adapting receptors (irritant receptors) or vagal C-fiber receptors (juxtacapillary receptors) close to pulmonary vessels (Böhrer et al., 1990). As reported by previous research studies, dezocine, as a mixed agonist–antagonist opioid, could activate κ receptors and antagonize the μ receptors to reduce OIC with no obvious adverse effects (Liu et al., 2015). Butorphanol, also as an agonist–antagonist opioid, could not only antagonize opioid-activated μ receptors but also activate the C-fiber receptor to inhibit the cough reflex afferent pathway (Zhang et al., 2018). Lidocaine was found to be effective in reducing OIC by suppressing brain stem function or anesthetizing the peripheral cough receptors (Poulton and James, 1979). Ketamine was reported to inhibit OIC by having an antagonistic effect on N-methyl-D-aspartate (NMDA) receptors (Said et al., 1995). Dexmedetomidine, as a highly selective α2-adrenergic agonist, could also reduce OIC via activating α2-adrenergic receptors to reverse muscular rigidity or relax tracheal smooth muscle contraction induced by histamine (Groeben et al., 2004).

In consistence with previously published studies, our results shed light on the effectiveness of five therapeutic drugs (lidocaine, ketamine, dezocine, butorphanol, and dexmedetomidine) in preventing OIC. Meta-analysis of RCTs conducted by Xiong et al. reported that dezocine could significantly reduce sufentanil-induced cough during general anesthesia induction, with no significant effect on vital signs (Xiong et al., 2020). Meta-analysis of RCTs conducted by Zhang et al. explained that butorphanol could also effectively prevent the incidence and severity of OIC (Zhang et al., 2018). Meta-analysis of RCTs conducted by Sun et al. suggested the effectiveness of prophylactic intravenous lidocaine in decreasing OIC during general anesthesia induction (Sun et al., 2014). Meta-analysis of RCTs conducted by Li et al. showed that prophylactic intravenous drugs such as ketamine, lidocaine, priming of fentanyl, dexmedetomidine, dezocine, and propofol was successful in inhibiting OIC (Shuying et al., 2016). Although all of these five drugs were effective in preventing OIC, they were compared with the placebo, and direct comparisons of different pharmacological interventions were absent. In this article, we not only compared the effectiveness of five drugs but also took advantage of network meta-analysis of RCTs to provide a hierarchy of these drugs.

As for adverse effects, a high dose of lidocaine could result in arrhythmia and cardiovascular depression during general anesthesia induction (Schlimp and Wiedermann, 2005). Ketamine could lead to hallucinations and elevation of blood pressure, intraocular pressure, and intracranial pressure (Shuying et al., 2016). Dexmedetomidine had adverse effects such as hypotension and bradycardia (Ebert et al., 2000). Currently, no significant effect on vital signs had been found in administration of dezocine. Dezocine and butorphanol could mainly result in respiratory depression, postoperative nausea, and vomiting (Sun et al., 2011; Zhang et al., 2018). Interestingly, we noticed that the combination of different drugs could effectively enhance the effect of reducing OIC. Honarmand et al. explained that a combination of ketamine and dexamethasone could significantly reduce the incidence of OIC than their single use (Honarmand et al., 2013). Saleh et al. found that ketamine in combination with dexmedetomidine could also effectively suppress OIC and delay the cough onset time (Saleh et al., 2014). Yu et al. revealed similar results in the combination of dexmedetomidine and midazolam for suppressing fentanyl-induced cough (Yu et al., 2012). Subsequent research studies should pay more attention to different drug combinations and their adverse effects.

In terms of the effects of different drug doses on OIC, Cheng et al. suggested that 0.03 mg/kg butorphanol was as effective as 0.015 mg/kg butorphanol in clinical practice to suppress fentanyl-induced cough (Cheng et al., 2016). Xu et al. revealed that dezocine attenuated fentanyl-induced cough in a dose-dependent manner, and the optimal dose was 0.1 mg/kg (Xu et al., 2015). Pandey et al. identified that the minimal dose of intravenous lidocaine for suppressing OIC was 0.5 mg/kg and any increased dose could not further reduce OIC (Pandey et al., 2005). Kim et al. found that a low dose of ketamine (0.1 mg/kg) was also effective in decreasing remifentanil-induced cough without influencing its severity and onset time (Kim et al., 2009). Zhou et al. also identified that the optimal dose of dexmedetomidine in the suppression of fentanyl-induced cough was 0.6 mg/kg, with no side effects (Zhou et al., 2019). In summary, the optimal dose of different drugs for preventing OIC needs to be fully explored.

As far as we are aware, this is the first network meta-analysis comparing the effectiveness of five therapeutic drugs (lidocaine, ketamine, dezocine, butorphanol, and dexmedetomidine) in preventing OIC, based on RCTs, which shall have a clear impact on the group baseline features and provide enough statistical power. Moreover, we not only conducted the direct comparisons of the five drugs but also performed indirect comparisons by means of network meta-analysis to provide a hierarchy of these drugs. Our analysis was anticipated to provide some references for guiding further clinical research. There were several limitations in this article too. First, the overall heterogeneity between different comparisons of drugs was low to moderate, except for butorphanol vs. placebo. Second, *p-*values of the node-splitting method between ketamine vs. lidocaine for incidence of OIC and mild severity of OIC were all below 0.05, indicating the inconsistency of the direct and indirect evidence. Third, due to the limitation of the meta-analysis, we could only use limited data obtained from previously published articles, and thus could not specify patients’ baseline characteristics and demographics. Hence, we currently faced difficulties in performing network meta-regression analyses to adjust for those effect modifiers and confounders. In summary, our results were merely analyzed in consideration of effectiveness, without consideration of different doses, adverse effects, time point of drug administration, and cost-benefit analysis. Subsequent high-quality RCTs were required to pay more attention to these aspects.

## Conclusion

Taken together, our results indicated that all of the five drugs, namely, lidocaine, ketamine, dezocine, butorphanol, and dexmedetomidine could prevent OIC for four clinical outcomes, compared with the placebo. Among them, dezocine had the best effect compared with that of other drugs. Moreover, the rank probability for the incidence of OIC, moderate severity of OIC, and severe severity of OIC from best to worst was dezocine, butorphanol, ketamine, dexmedetomidine, lidocaine, and placebo, and the rank probability for mild severity of OIC from first to last was dezocine, butorphanol, ketamine, lidocaine, dexmedetomidine, and placebo, based on the network meta-analysis results. Our analysis was anticipated to provide some references for guiding further clinical research, and subsequent high-quality RCTs were required to verify our results.

## Data Availability

The raw data supporting the conclusions of this article will be made available by the authors, without undue reservation.
