# Supplementary figures and images for: Comparison of Five Prophylactically Intravenous Drugs in Preventing Opioid-Induced Cough: A Bayesian Network Meta-Analysis of Randomized Controlled Trials
Source: Front Pharmacol. 2021 Nov 17;12:684276. doi: 10.3389/fphar.2021.684276 (PMC8635493; doi:10.3389/fphar.2021.684276)

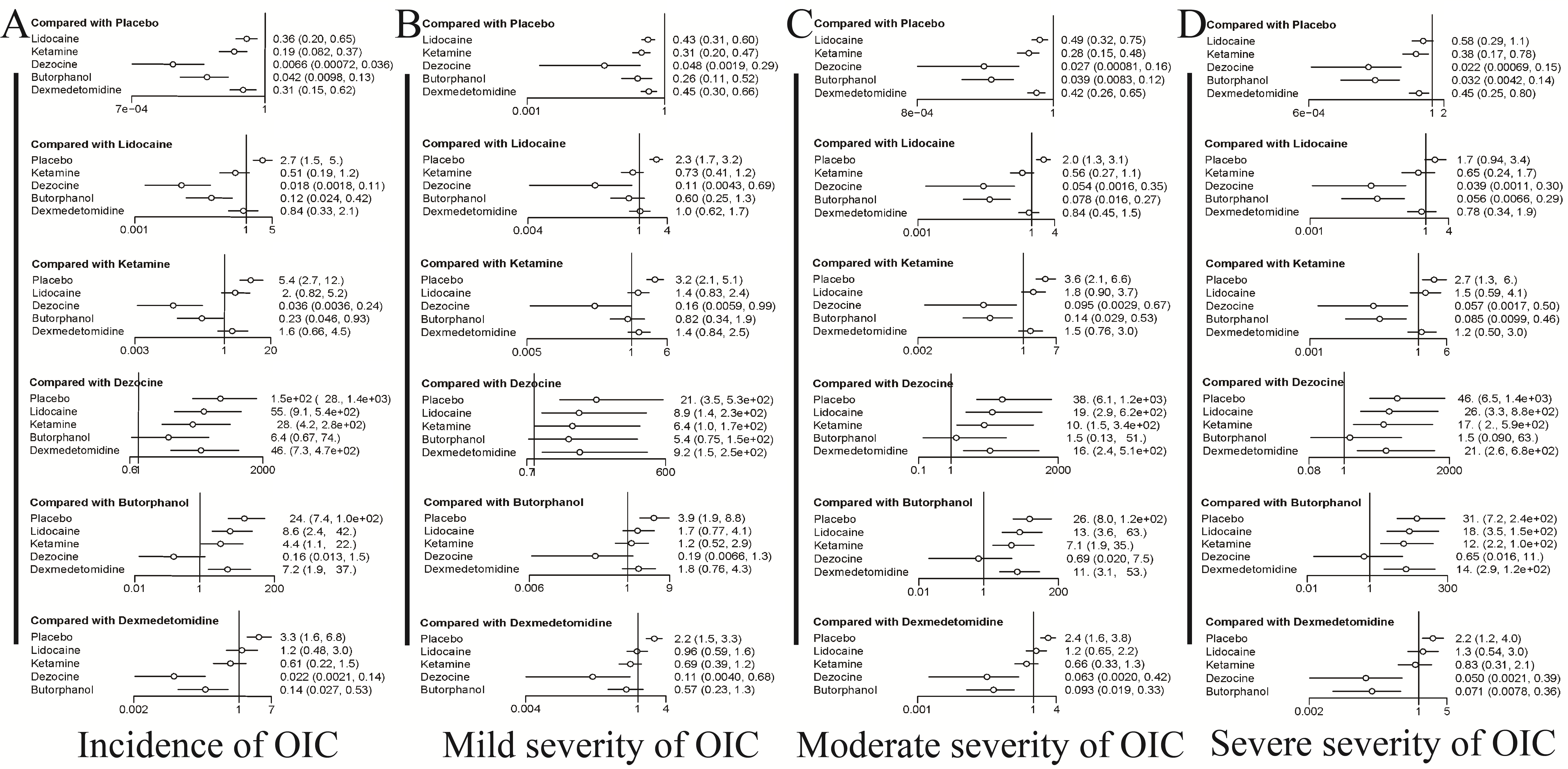

Supplement: Supplementary file 1 [file Image3.TIF]

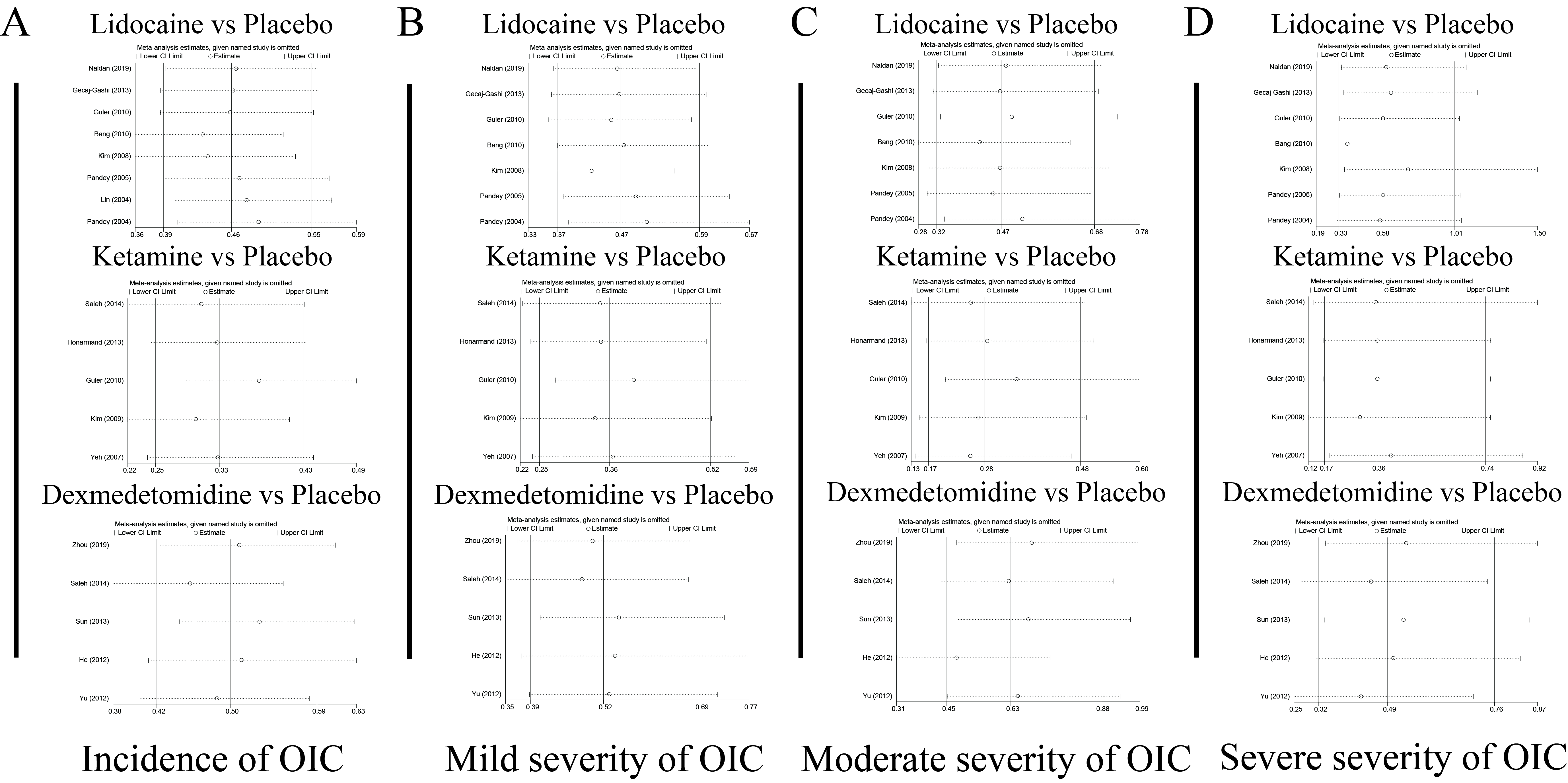

Supplement: Supplementary file 2 [file Image4.TIF]

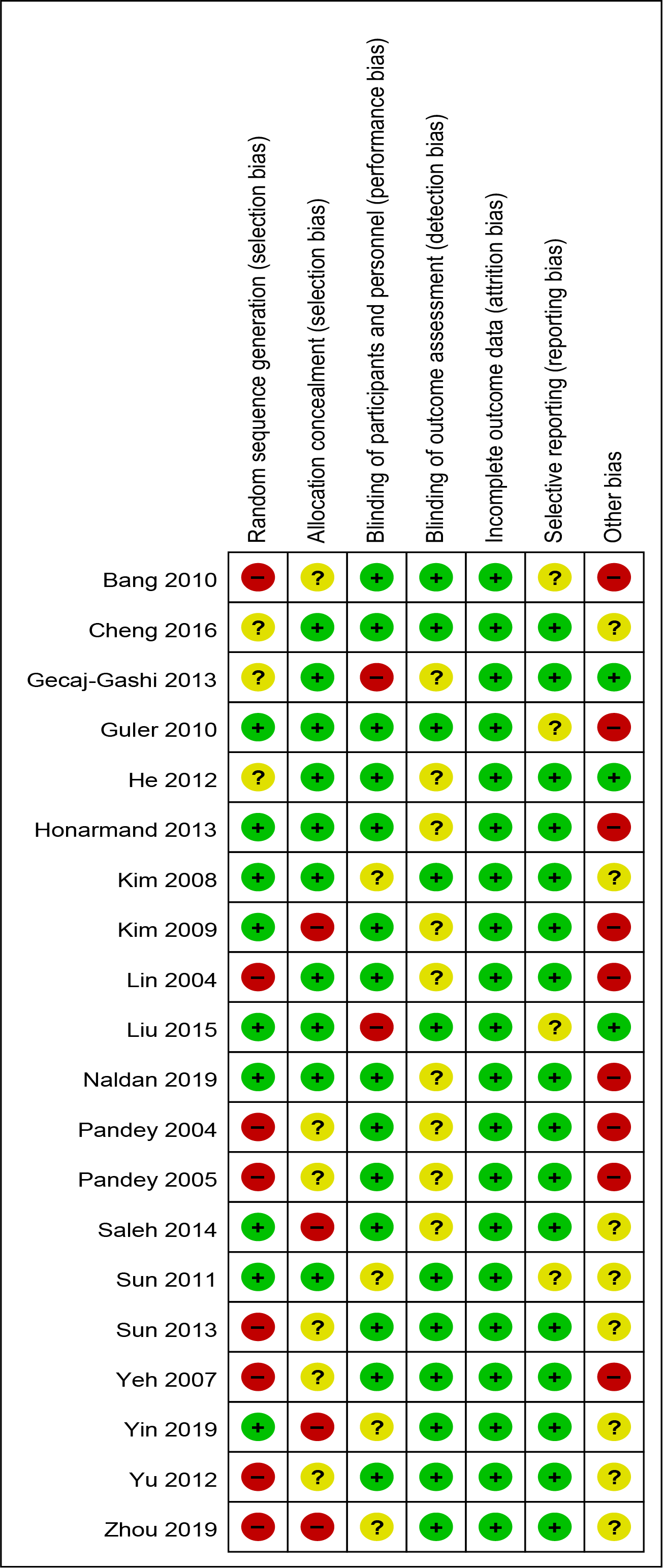

Supplement: Supplementary file 3 [file Image2.TIF]

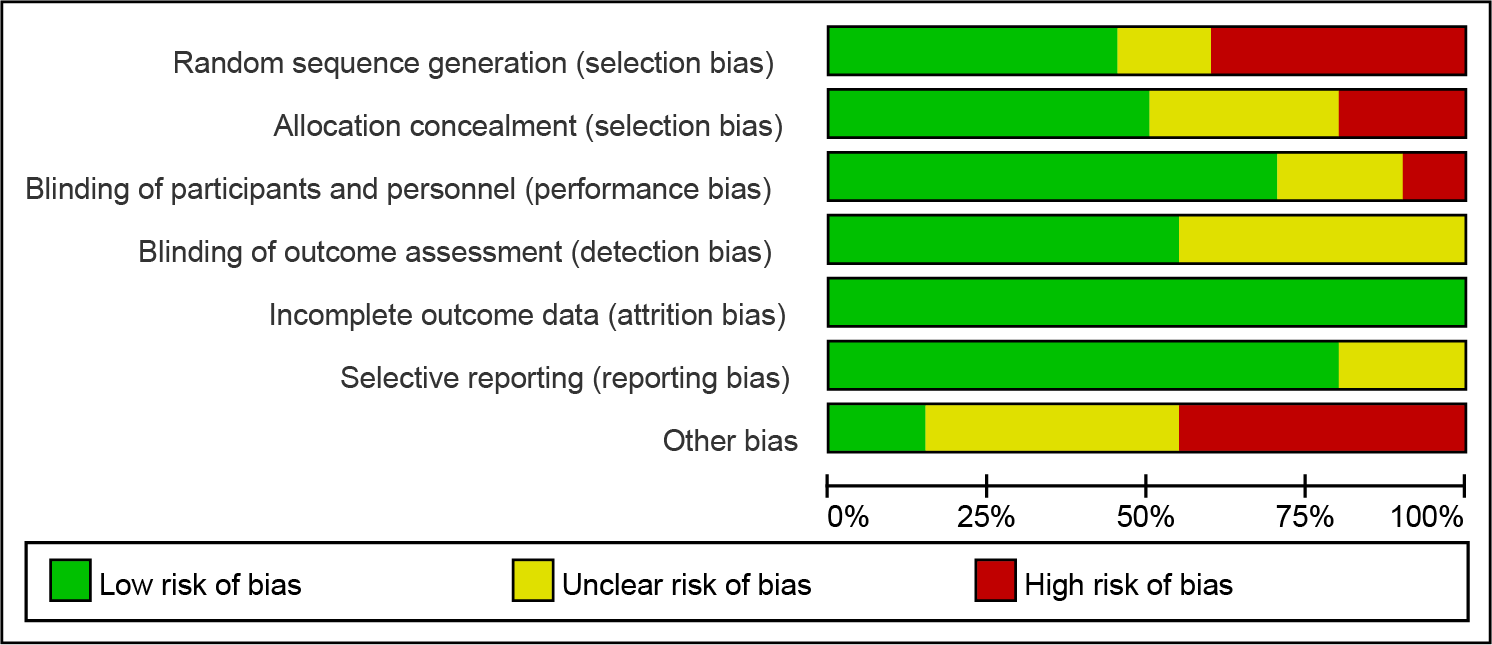

Supplement: Supplementary file 4 [file Image1.TIF]
