# Supplementary material for: Comparison of Five Prophylactically Intravenous Drugs in Preventing Opioid-Induced Cough: A Bayesian Network Meta-Analysis of Randomized Controlled Trials
Source: Front Pharmacol. 2021 Nov 17;12:684276. doi: 10.3389/fphar.2021.684276 (PMC8635493; doi:10.3389/fphar.2021.684276)
Supplement: Supplementary file 5 [file DataSheet2.DOCX]

**Table S2**. PRISMA 2020 for abstracts checklist;

| Section and topic | Item # | Checklist item |  |
| --- | --- | --- | --- |
| Title |  |  |  |
| Title | 1 | Identify the report as a systematic review. | Page 1 |
| Background |  |  |  |
| Objectives | 2 | Provide an explicit statement of the main objective(s) or question(s) the review addresses. | Page 2 |
| Methods |  |  |  |
| Eligibility criteria | 3 | Specify the inclusion and exclusion criteria for the review. | Page 2 |
| Information sources | 4 | Specify the information sources (e.g. databases, registers) used to identify studies and the date when each was last searched. | Page 2 |
| Risk of bias | 5 | Specify the methods used to assess risk of bias in the included studies. | Page 2 |
| Synthesis of results | 6 | Specify the methods used to present and synthesise results. | Page 2 |
| Results |  |  |  |
| Included studies | 7 | Give the total number of included studies and participants and summarise relevant characteristics of studies. | Page 2 |
| Synthesis of results | 8 | Present results for main outcomes, preferably indicating the number of included studies and participants for each. If meta-analysis was done, report the summary estimate and confidence/credible interval. If comparing groups, indicate the direction of the effect (i.e. which group is favoured). | Page 2 |
| Discussion |  |  |  |
| Limitations of evidence | 9 | Provide a brief summary of the limitations of the evidence included in the review (e.g. study risk of bias, inconsistency and imprecision). | Page 2 |
| Interpretation | 10 | Provide a general interpretation of the results and important implications. | Page 2 |
| Other |  |  |  |
| Funding | 11 | Specify the primary source of funding for the review. | Page 11 |
| Registration | 12 | Provide the register name and registration number. | Page 4 |
